# The effect of 1% glucose loading on metabolism in the elderly patients during remifentanil-induced anesthesia: a randomized controlled trial

**DOI:** 10.1186/s12871-020-01061-3

**Published:** 2020-06-06

**Authors:** Kohei Fukuta, Asuka Kasai, Noriko Niki, Yuki Ishikawa, Ryosuke Kawanishi, Nami Kakuta, Yoko Sakai, Yasuo M. Tsutsumi, Katsuya Tanaka

**Affiliations:** 1 Department of Anesthesiology, Graduate School of Biomedical Sciences, Tokushima University, 3-18-15 Kuramoto, Tokushima, 770-8503 Japan; 2 Department of Anesthesiology and Critical Care, Graduate School of Biomedical and Health Sciences, Hiroshima University, 1-2-3 Kasumi Minami, Hiroshima, 774-8551 Japan

**Keywords:** Glucose, Metabolism, Elderly, Remifentanil

## Abstract

**Background:**

Previous studies showed that remifentanil-induced anesthesia can inhibit surgical stress response in non-diabetic adult patients and that low-dose glucose loading during anesthesia may attenuate fat catabolism. However, little is known about the influence of glucose loading on metabolism in elderly patients, whose condition may be influenced by decreased basal metabolism and increased insulin resistance. We hypothesized that, in elderly patients, intraoperative low glucose infusion may attenuate the catabolism of fat without causing harmful hyperglycemia during remifentanil-induced anesthesia.

**Methods:**

Elderly, non-diabetic patients scheduled to undergo elective surgery were enrolled and randomized to receive no glucose (0G group) or low-dose glucose infusion (0.1 g/kg/hr. for 1 h followed by 0.05 g/kg/hr. for 1 h; LG group) during surgery. Glucose, adrenocorticotropic hormone (ACTH), 3-methylhistidine (3-MH), insulin, cortisol, free fatty acid (FFA), creatinine (Cr), and ketone body levels were measured pre-anesthesia, 1 h post-glucose infusion, at the end of surgery, and on the following morning.

**Results:**

A total of 31 patients (aged 75–85) were included (0G, *n* = 16; LG, *n* = 15). ACTH levels during anesthesia decreased significantly in both groups. In the LG group, glucose levels increased significantly after glucose loading but hyperglycemia was not observed. During surgery, ketone bodies and FFA were significantly lower in the LG group than the 0G group. There were no significant differences in insulin, Cr, 3-MH, and 3-MH/Cr between the two groups.

**Conclusion:**

Remifentanil-induced anesthesia inhibited surgical stress response in elderly patients. Intraoperative low-dose glucose infusion attenuated catabolism of fat without inducing hyperglycemia.

**Trial registration:**

This study has been registered with the University hospital Medical Information Network Center (http://www.umin.ac.jp/english/). Trial registration number: UMIN000016189. The initial registration date: January 12th 2015.

## Background

Glucose tolerance is decreased during surgery by catecholamines and stress hormones, such as cortisol and adrenocorticotropic hormone (ACTH) [1–4], and intraoperative hyperglycemia is a risk factor for postoperative complications and mortality [5–7]. Therefore, glucose solution is not generally infused during surgery, despite the fact that an energy deficit may lead to the catabolism of fats and/or proteins. Several studies show that remifentanil reduces the stress response during surgery [8–11]. We previously reported that anesthesia using remifentanil limits the surgical stress response in non-diabetic adult patients and that low-dose glucose loading during anesthesia may attenuate the catabolism of fat [12]. Sawada et al. also showed that intraoperative glucose infusion suppressed lipolysis and proteolysis in patients anesthetized with remifentanil [13].

It is reported that basal energy expenditure (EE) is negatively associated with age in subjects > 52 years old [14]. In addition, many studies have reported that insulin secretion decreasing and resistance increasing with age [15–18]. Elderly patients are, therefore, influenced by a decrease in basal metabolism and an increase in insulin resistance. These studies suggest that, while low-dose glucose loading during remifentanil-induced anesthesia may decrease stress hormone secretion and fat catabolism without causing hyperglycemia in adults, it may induce hyperglycemia in elderly patients. Currently, little is known about the effect of glucose loading on metabolism in elderly patients during remifentanil-induced anesthesia.

Here, we hypothesized that, in elderly patients, intraoperative low glucose infusion during remifentanil-induced anesthesia may attenuate the catabolism of fat without causing harmful hyperglycemia. To test this hypothesis, we examined the effects of glucose infusion on metabolism in elderly patients anesthetized with remifentanil.

## Methods

### Study design and patient selection

Elderly (aged 75–85 years), non-diabetic patients scheduled to undergo elective surgery in the Tokushima University Hospital between September 2015 and September 2016 were enrolled. Patients were required to have an American Society of Anesthesiologists physical status of 1 or 2 and a scheduled surgery duration of > 1 h. Obese (body mass index [BMI] > 30 kg/m^2^) and emaciated (BMI < 17 kg/m^2^) patients were excluded, as were those taking steroids or diagnosed with diabetes or thyroid disease. Patients requiring the use of a tourniquet or laparoscopy during surgery were also excluded from the final analysis. Homeostasis Model Assessment Insulin Resistance (HOMA-IR) was calculated by the formula: fasting insulin [μIU/ml] × fasting glucose [mg/dl] / 405. Eligible patients were randomized to receive no glucose (0G group) or a low-dose glucose infusion (0.1 g/kg/hr. for 1 h followed by 0.05 g/kg/hr.; LG group) during surgery.

The study was approved by Clinical Trial Center For Developmental Therapeutics of the Tokushima University Hospital, and the reference number was 2211–1. All participating subjects provided written informed consent. The study was registered with the University hospital Medical Information Network Center (http://www.umin.ac.jp/english/); ID: UMIN000016189.

### Anesthesia management and study protocol

Patients were allowed to eat until 00:00 h on the day of surgery. Patients scheduled for surgery in the morning received 250 ml (200 kcal) of Arginaid Water® (Nestle Japan Ltd., Tokyo, Japan), and those scheduled in the afternoon received 500 ml (400 kcal) 2 h before anesthesia. The nutrient profiles of Arginaid Water® are shown in Table 1. No patients were premedicated.

**Table 1:** Major nutrients in Arginaid Water®

Nutrients	Arginaid water 100 mL
Calories (kcal)	80
Moisture (g)	85.6
Arginine (g)	2.0
CHO (g)	18
Fat (g)	0
Sodium (mg)	0
Phosphate (mg)	140
Zinc (mg)	0.8
Copper (mg)	0.8

On arrival in the operating room, a 20 G catheter was inserted into the forearm of each patient and bicarbonate Ringer’s solution without glucose was infused. General anesthesia was induced by intravenous administration of thiamylal (3 mg/kg) and remifentanil (0.25–0.5 μg/kg/min), and maintained with sevoflurane (end-tidal sevoflurane ≥1.0%) and remifentanil (0.2–0.5 μg/kg/min). Muscle relaxation with 0.7 mg/kg rocuronium bromide was performed to facilitate tracheal intubation. Rocuronium bromide was administered intermittently, if required. All patients were maintenance of the BIS value between 40 to 60 during the surgery.

**Fig. 1:**
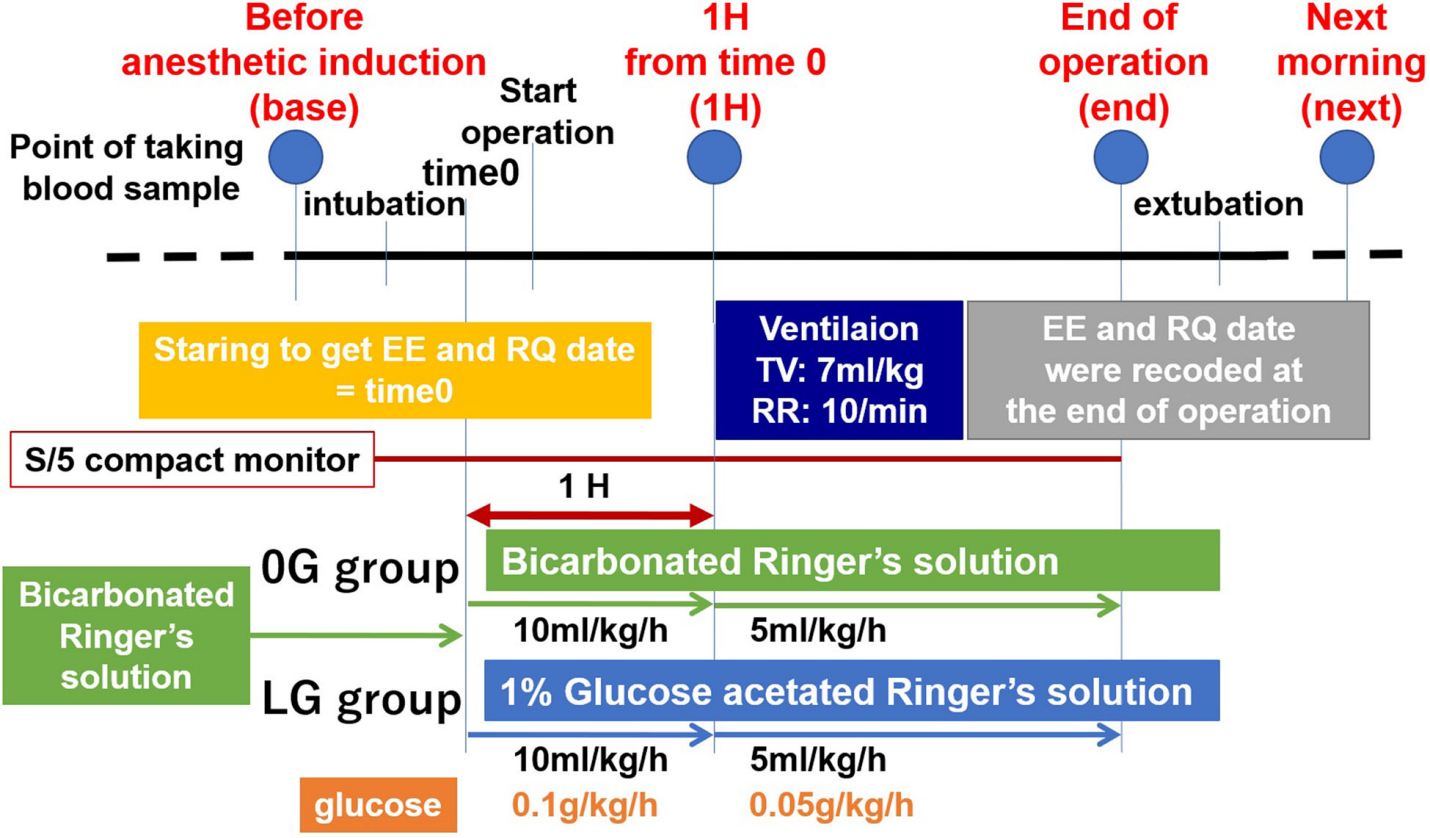
Study protocol. EE, energy expenditure; RQ, respiratory quotient; TV, tidal volume; RR, respiratory rate

The study protocol is illustrated in Fig. 1. After tracheal intubation, the heat and moisture exchange filter was equipped with an S/5 compact monitor (GE Healthcare, Helsinki, Finland). The tidal volume was set at 7 ml/kg, the respiratory rate set with the aim of normocapnia, and the O_2_/air mixture at FiO_2_ 0.5. Stable data for respiratory quotient (RQ), oxygen consumption ($\dot{\mathrm{V}}$ O_2_), carbon dioxide output ($\dot{\mathrm{V}}$ CO_2_), and EE were obtained from the S/5 compact monitor within approximately 20 min (time 0). In the LG group, the solution was switched to 1% glucose acetated Ringer’s solution at time 0, with the solution for both groups being initiated at 10 ml/kg/hr. for 1 h, followed by 5 ml/kg/hr. Therefore, in the LG group, glucose was taken at 0.1 g/kg/hr. for 1 h, followed by 0.05 g/kg/hr.

### Measurements

Blood was sampled at baseline (introduction of anesthesia), 1 h after time 0, at the end of surgery, and the next morning. Glucose concentrations were measured using a blood glucose monitor (Medisafe Fit, TERUMO, Tokyo, Japan). Blood glucose concentrations > 200 mg/dl were defined as hyperglycemia in this study. Patients with blood glucose concentrations > 250 mg/dl were intravenously injected with 2 U insulin. Blood samples were centrifuged at 150 g at 4 °C for 10 min (Table Top cooling centrifuge 2800, Kubota, Tokyo, Japan), and plasma and serum samples were stored at − 20 °C until analysis. Plasma concentrations of glucose, ACTH, and 3-methylhistidine (3-MH) and serum concentrations of insulin, cortisol, free fatty acid (FFA), creatinine (Cr), and ketone bodies were analyzed. Plasma glucose was measured using the hexokinase method, plasma ACTH by an electro chemiluminescent immunoassay, and plasma 3-MH by high-performance liquid chromatography. Serum concentrations of FFA, ketone bodies, and Cr were measured enzymatically, serum insulin by a chemiluminescent enzyme immunoassay, and serum cortisol by a radioimmunoassay. RQ, $\dot{\mathrm{V}}$ O_2_, $\dot{\mathrm{V}}$ CO_2_, and EE were measured by the S/5 compact monitor and recorded at 30 min intervals until the end of surgery.

### Randomization and blinding

We used a computer-generated distribution (QuickCalcs, GraphPad Inc., La Jolla, CA, USA) for randomly allocation. Anesthesiologists collecting intraoperative data were not blinded to group assignment, however, patients, surgeons and another anesthesiologist evaluating the date were blinded to group assignment.

### Endpoints

The primary endpoint in the present study was the concentration of FFA. Secondary endpoints included the concentration of ketone bodies (i.e. lipid metabolism), the value of 3MH/Cr (i.e. protein catabolism), glucose and serum insulin concentrations (i.e. glucose metabolism), ACTH and serum cortisol concentrations (i.e. stress hormone), and RQ (i.e. the energy source that they used).

### Statistical analysis

The primary endpoint was the concentration of FFA. Our previous study reported that the concentration of FFA in adult patients at 2 h after the initiation of infusion was significantly higher in patients who received no intraoperative glucose than in those who received 1% glucose intraoperatively (840 ± 290 versus 510 ± 240 μEq/L, respectively) [12]. Anticipating that the mean difference between the groups during surgery would be 330 ± 260 μEq/L, the minimum number of patients in each group was 12, with an alpha of 0.05 and a power of 80% for FFA. We estimated that 32 patients should be included in this trial, with 16 patients in each group, because of possible dropouts and complications during surgery.

The Shapiro–Wilk test for fit with normal distribution was performed. In parametric data, differences between the time points within each group were compared using repeated-measures analysis of variance with the Bonferroni post hoc test. Parametric data at the same time points between the two groups of variables were analyzed by unpaired t-tests. In nonparametric data, differences between the time points within each group were compared using Friedman’s test. Nonparametric data at the same time points between the two groups of variables were analyzed by the Mann–Whitney rank-sum test. Nominal scales were analyzed by the Chi-squared test. *P* < 0.05 was considered to be statistically significant. All statistical analyses were performed with SPSS version 20 (IBM, New York, NY, USA).

## Results

**Fig. 2:**
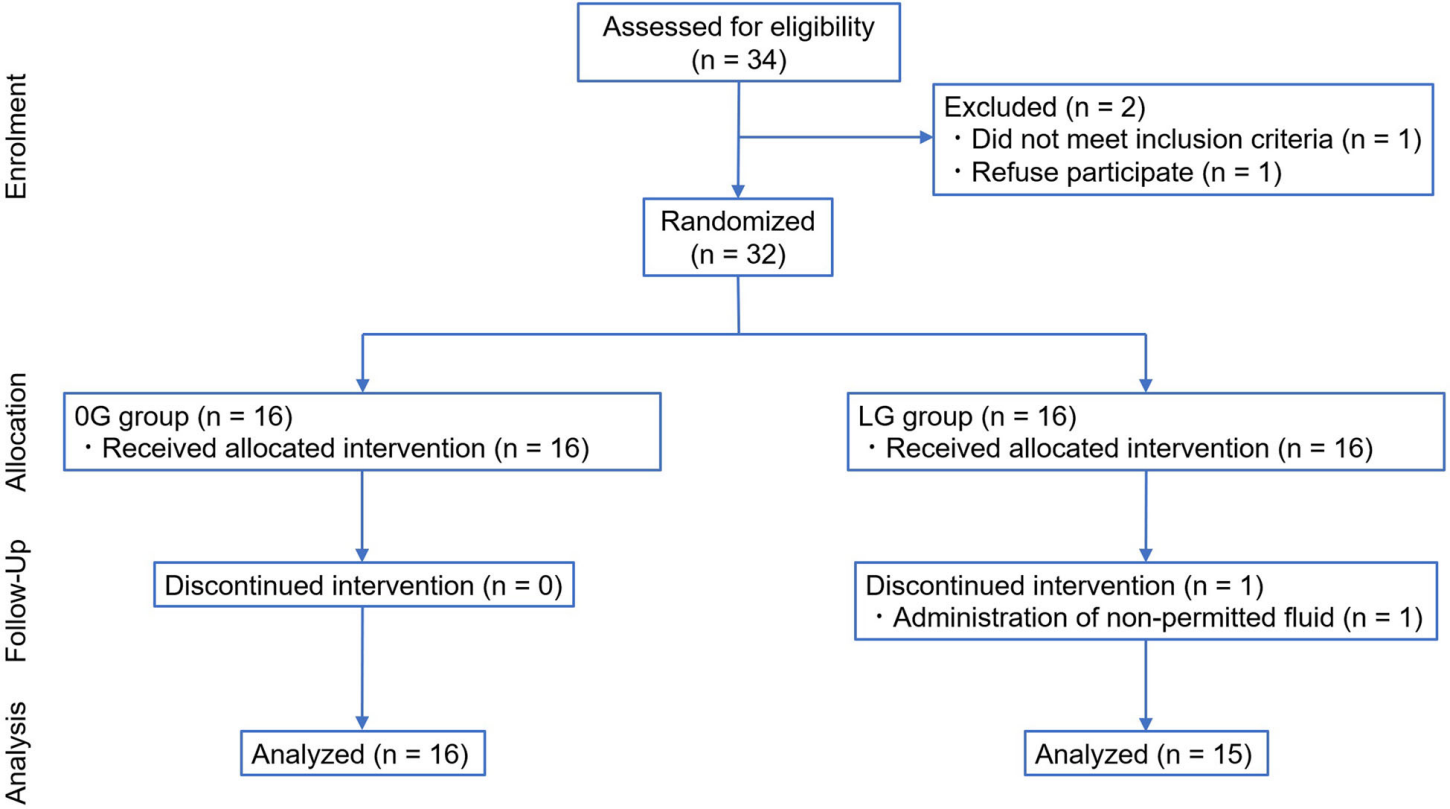
Study flow diagram

Patient recruitment and flow through the protocol is summarized in Fig. 2. Although 34 patients were enrolled, one was excluded due to undiagnosed diabetes and one patient refused to participate. Thirty-two patients were randomized to the 0G and LG groups; one patient in the LG group was excluded from the analysis as a non-permitted fluid was administered to treat bleeding. Therefore, 31 patients completed the trial and were included in the analysis. The patients’ demographic data and surgical procedures are shown in Tables 2 and 3. No notable differences were seen between the 0G and LG groups.

**Table 2:** Demographic data

	0G group	LG group	*P* value
Male/Female	4/12	7/8	*P* = 0.21
Age (yr)	78.5 ± 2.8	79.0 ± 4.0	*P* = 0.83
Height (cm)	151.0 ± 6.3	157.7 ± 7.6	*P* = 1.0
Weight (kg)	57.3 ± 6.7	58.8 ± 13.1	*P* = 0.70
BMI (kg/m^2^)	24.0 ± 2.0	24.2 ± 3.1	*P* = 0.81
HOMA-IR	1.85 ± 0.95	1.61 ± 0.94	*P* = 0.40
APACHE II score	8 ± 4	7 ± 2	*P* = 4.23
Operation time (min)	101.5 ± 35.9	142.0 ± 69.4	*P* = 0.06
Blood loss (ml)	50.0 ± 47.1	90.0 ± 267.5	*P* = 0.57

Data are expressed as the mean ± SD

There were no statistically significant differences between the 2 groups

**Table 3:** Types of surgical procedure performed

0G group		LG group	
Cervical laminoplasty	3	Cervical laminoplasty	1
Lumbar partial laminectomy	1	Microendscopic lunbar laminectomy	1
Discectomy	1	Lumbar posterior fusion	3
Total hip arthroplasty	2	Extreme lateraI interbody fusion	1
Mastectomy	3	Total hip arthroplasty	1
Skin malignant tumor resection	1	Mastectomy	1
Scar plasty	1	Patial mastectomy	1
Laryngomicrosurgery	1	Flap surgery	1
Dacryocystorhinostomy	1	Laryngomicrosurgery	1
Perineoplasty	1	Endoscopic sinus surgery	1
Adnexectomy+colpoplasty	1	Dacryocystorhinostomy	1
		Tension-free vaginal tape	1
		Closure of colostomy	1

**Fig. 3:**
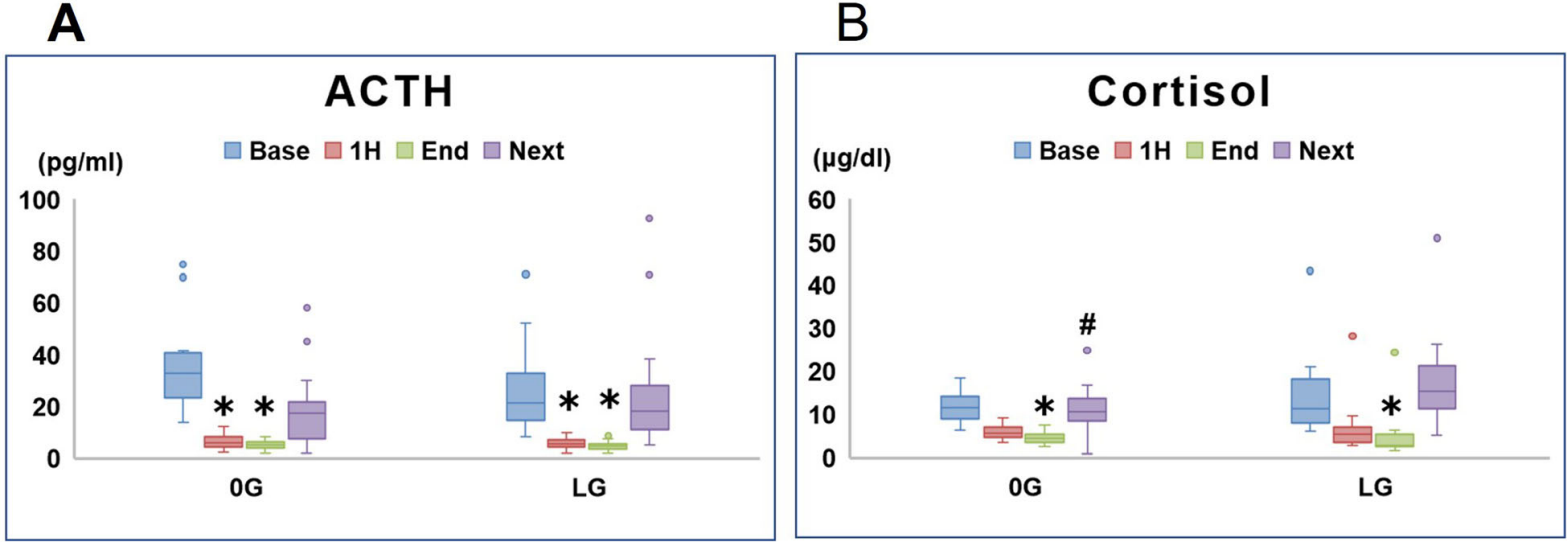
Plasma ACTH (**a**) and serum cortisol (**b**) concentrations in the 0G and LG groups prior to induction of anesthesia (base), at 1 h (1H) from time 0, at the end of surgery (end), and on the next morning (next). *P < 0.05 versus baseline; #*P* < 0.05 between groups

Levels of ACTH and cortisol at each study timepoint are shown in Fig. 3. ACTH levels during surgery were significantly lower than baseline in both groups (Fig. 3a).

**Fig. 4:**
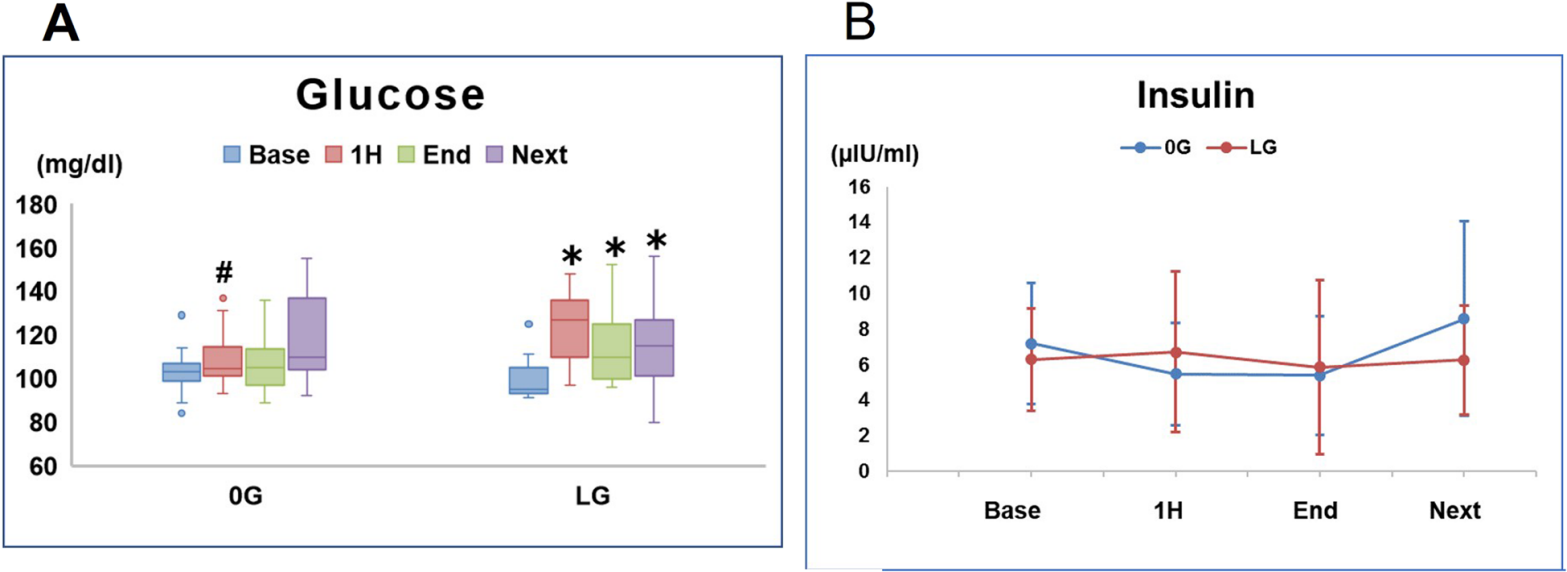
Plasma glucose (**a**) and serum insulin (**b**) concentrations in the 0G and LG groups prior to induction of anesthesia (base), at 1 h (1H) from time 0, at the end of surgery (end), and on the next morning (next). *P < 0.05 versus baseline; #*P* < 0.05 between groups

As shown in Fig. 4a, plasma glucose levels in the LG group were significantly higher than those in the 0G group at 1 h (*P* = 0.006). At 1 h and at the end of surgery, plasma glucose levels were significantly higher than baseline levels in the LG group (1 h vs baseline: *P* < 0.001, the end of surgery vs baseline: *P* = 0.043). However, the highest glucose concentration in the LG group was 156 mg/dl and none of the patients in either group required intravenous insulin or experienced hypoglycemia (< 70 mg/dl).

**Fig. 5:**
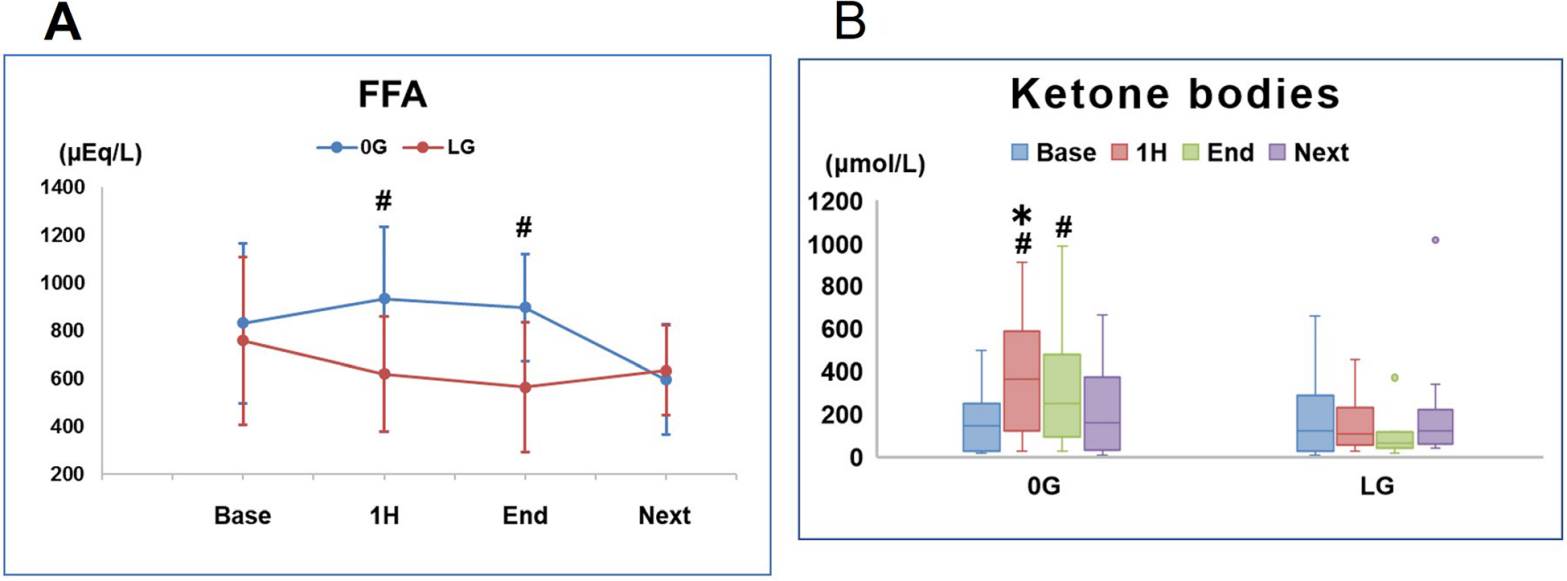
Serum FFA (**a**) and ketone body (**b**) concentrations in the 0G and LG groups prior to induction of anesthesia (base), at 1 h (1H) from time 0, at the end of surgery (end), and on the next morning (next). *P < 0.05 versus baseline; #*P* < 0.05 between groups

FFA levels in the LG group were significantly lower than those seen in the 0G group at 1 h and the end of surgery (1 h: *P* = 0.004, the end of surgery: *P* = 0.001; Fig. 5a). Levels of ketone bodies in the LG group were significantly lower than those in the 0G group at 1 h and at the end of surgery (1 h: *P* = 0.037, the end of surgery: *P* = 0.007; Fig. 5b). Levels of ketone bodies at 1 h were significantly higher than those at baseline in the 0G group (*P* = 0.02; Fig. 5b).

**Fig. 6:**
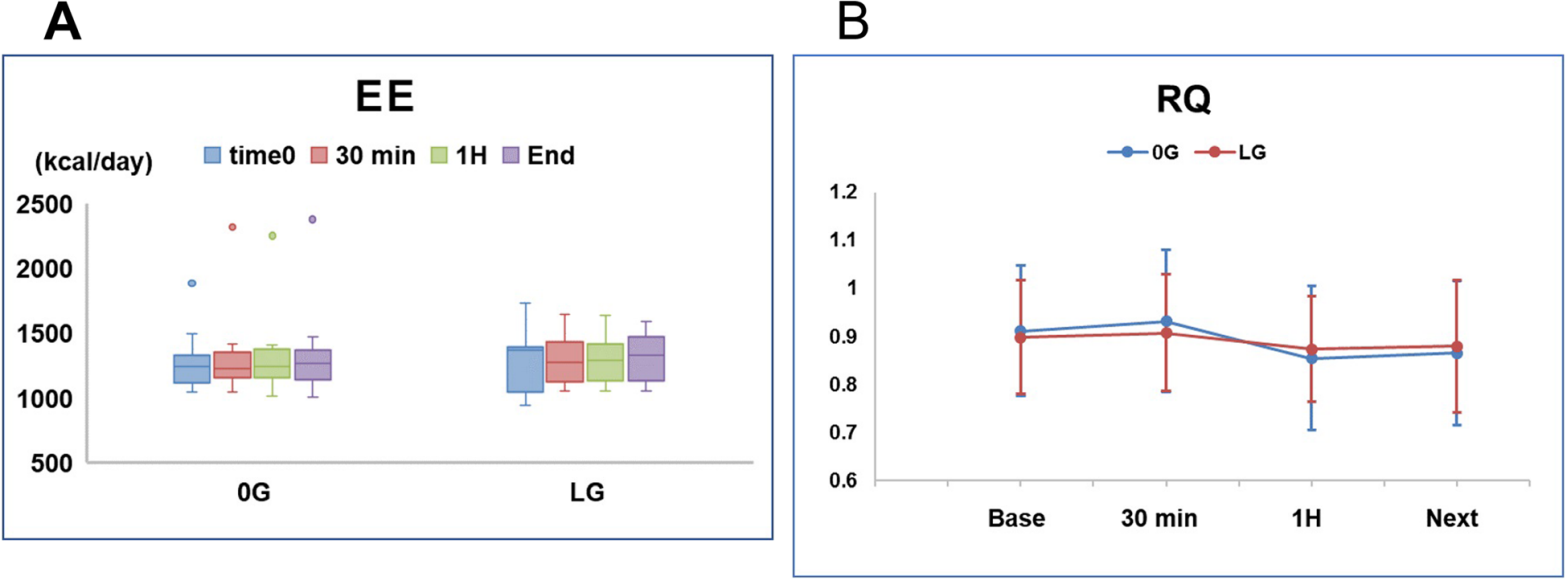
EE (**a**) and RQ (**b**) in the 0G and LG groups from the time of stabilization (time 0) to the end of surgery (end). EE, energy expenditure; RQ, respiratory quotient

There were no significant differences between the two groups in EE (Fig. 6a), RQ (Fig. 6b), $\dot{\mathrm{V}}$ O_2_, $\dot{\mathrm{V}}$ CO_2_, insulin (Fig. 4b), Cr, 3-MH, and 3-MH/Cr.

## Discussion

In this study of elderly patients, FFA levels in the LG group were significantly lower than those in the 0G group at 1 h and at the end of surgery. Levels of ketone bodies in the LG group were significantly lower than those seen in the 0G group at 1 h and the completion of surgery. In addition, levels at 1 h were significantly higher than those seen at baseline in the 0G group. These results indicate that 1% glucose loading during remifentanil-induced anesthesia attenuated the catabolism of fat in this patient group.

Our previous study of adult patients demonstrated that ketone levels in subjects with no glucose loading were approximately 300 μmol/L during surgery [12], whereas in the current study of elderly patients, ketone body levels were approximately 500 μmol/L in the 0G group. However, in both studies, ketone body levels during surgery were approximately 200 μmol/L in the LG group. This observation suggests that low-dose glucose loading may be more effective in elderly patients than in adult patients as a whole.

In the present study, all patients were given Arginaid Water® 2 h before anesthesia. Arginaid Water® are carbohydrate with aminoacid solution, and the nutrient profiles are shown in Table 1. In our previous study, we investigated whether the intake of preoperative carbohydrate with aminoacid solution can improve starvation status and lipid catabolism before the induction of anesthesia, and reported that the intake of preoperative carbohydrate with aminoacid solution significantly decrease FFA and ketone bodies at the initiation of anesthesia compared with the control group [19]. Therefore, non-preoperative Arginaid Water® may cause more significantly difference in FFA and ketone bodies between 0G group and LG group.

HOMA-IR has been used widely to measure insulin sensitivity and resistance based on fasting plasma glucose and insulin concentrations [20, 21]. Esteghamati A et al. reported that the 75th percentile of HOMA-IR was 1.6 in heaIthy Iranians [22]. Beak JH et al. reported that the overall optimal cut-off value of HOMA-IR for identifying dysglycemia was 1.6 in both sex, and that the cut-off values for type 2 diabetes mellitus were 2.87 in men and 2.36 in women [23]. Ascaso JF et al. reported that the 75th percentile value as the cut-off point to define insulin resistance corresponded with a HOMA-IR of 2.6 [24]. In the present study, HOMA-IR did not differ significantly between the two groups (0G group: 1.85 ± 0.95, LG group: 1.61 ± 0.94, *P* = 0.40; Table 2). The highest HOMA-IR in the 0G group was 4.0, and that in the LG group was 4.2. Therefore, in the present study, insulin resistance were increased in both groups.

In the present study, no significant changes in insulin levels were seen. However, our previous study of adult patients showed a significant increase in insulin levels after low-dose glucose loading during surgery [12]. Many studies have reported that insulin secretion decreasing and resistance increasing with age [15–18]. Lozzo et al. investigated basal β-cell function in 957 non-diabetic European patients aged 18–85 years, reporting that aging is associated with decreased basal insulin release [15]. Muzumdar et al. studied insulin secretion in rats aged 3–20 months, showing that glucose-stimulated insulin secretion decreased with age in this in vivo model [16]. Reaven et al. studied glucose-stimulated insulin release in β-cells of 2–18-month-old rats, demonstrating that the aging process leads to defects in glucose-stimulated insulin release from β-cells [25]. Therefore, the results seen in our current and previous studies support the data obtained by other groups indicating that aging is associated with impaired glucose-stimulated insulin release.

Parsons et al. reported that acute hyperglycemia adversely affects stroke outcome [26]. In the present study, plasma glucose levels were higher in the LG group than in the 0G group at 1 h and higher at 1 h and at the end of surgery than at baseline in the LG group. However, as the highest concentration of glucose in both groups was 156 mg/dl, none of the patients required intravenous insulin. These results suggest that, even in elderly patients, remifentanil-induced anesthesia may prevent hyperglycemia associated with low-dose glucose infusion.

Remifentanil-induced anesthesia decreases stress hormones, such as ACTH and cortisol, and suppresses the surgical stress response in adults [8–11]. Demirbilek et al. compared the effects of remifentanil and alfentanil as part of total intravenous anesthesia on plasma concentrations of cortisol, insulin, and glucose in patients undergoing abdominal hysterectomy, demonstrating that remifentanil-induced anesthesia was associated with decreased cortisol levels [8]. We previously reported that anesthesia using remifentanil significantly decreases ACTH and cortisol levels in adult patients [12]. In the present study, ACTH in both the 0G and LG groups was significantly decreased during remifentanil-induced anesthesia, suggesting that general anesthesia using remifentanil may suppress the stress response in elderly patients.

There were no significant differences in 3-MH/Cre levels in our current or previous studies [12]. In the present study, the surgical procedures were primarily performed on the body surface, rather than being highly invasive, and the surgery time was approximately 2 h. The aim was to exclude the influence of surgical stress in order to observe the effect of aging in the present study. Sawada et al. showed that 3-MH/Cr levels at 6 h were significantly higher than levels prior to anesthesia during major surgery in patients receiving no glucose infusion [13]. This discrepancy suggests that significant surgical stress may induce protein catabolism in the absence of glucose loading.

In the present study, subjects were elderly, but were not obese. It is reported that age per se does not increase HOMA-IR levels and that the changes might be related to higher rates of obesity in older subjects [27]. In addition, it is reported that the deterioration of glucose tolerance in healthy elderly subjects is due to a decrease in insulin secretion and can be explained by the degree of obesity rather than age [28]. Furthermore, one study demonstrated that aging has no effect on insulin sensitivity independent of changes in body composition [29]. These studies show that age itself does not increase insulin resistance and that changes may be related to the development of obesity. The low-dose glucose infusion during remifentanil-induced anesthesia in obese patients may, therefore, induce hyperglycemia. In the present study, one patient whose BMI was > 30 kg/m^2^ was excluded and BMI did not differ significantly between the two groups (0G group: 24.0 ± 2.0 kg/m^2^, LG group: 24.2 ± 3.1 kg/m^2^, *P* = 0.81; Table 2). The highest BMI in the 0G group was 26.9 kg/m^2^, and that in the LG group was 29.1 kg/m^2^. While further studies are required to evaluate this association further, in the current study of elderly, non-obese patients, low-dose glucose loading during remifentanil-induced anesthesia attenuated fat catabolism without causing hyperglycemia.

Acute Physiology and Chronic Health Evaluation (APACHE) II score is a general measure of severity of disease, has been used to predict hospital mortality [30–32]. We have done analysis to evaluate APACHE II score with preoperative data. Gupta S et al. reported that critically ill patients (CIP) with APACHE II score of ≥15 at admission or within 24 h are at risk for the development of CIP [31]. Joe BH et al. reported that patients with APACHE II score greater than 20 had tendency to expire than the others, and that APACHE II score more than 20, rather than cardiac function, is associated with mortality in patients with stress-induced cardiomyopathy [32]. In present study, APACHE II score did not differ significantly between the two groups (0G group: 8 ± 4, LG group: 7 ± 2, *P* = 4.23; Table 2). The highest APACHE II score in the 0G group was 12, and that in the LG group was 11. In present study, elderly (75–85 years) were required to have American Society of Anesthesiologists physical status of 1 or 2. Therefore, in present study, there were low severity in both groups.

In the present study, we used sevoflurane, but not propofol, to maintain the general anesthesia in elderly patients. Sevoflurane and propofol are commonly used general anesthetics during surgery. Several clinical studies were tried to see whether the choice of the anesthetic agent make a difference in postoperative delirium or the postoperative cognitive dysfunction (POCD) after non-cardiac surgery in elderly patients. Some studies indicated that propofol reduced POCD as compared with sevoflurane [33, 34]. In contrast, it was reported that propofol significantly increased the delirium rating scale on day 2 and 3 after surgery, the time required for emergence from anesthesia as defined by eye opening and the time to tracheal extubation, as compared with sevoflurane [35]. Recent systematic review reported that it was uncertain whether maintenance with propofol or with volatile anesthetics affect incidence of postoperative delirium, mortality, or length hospital stay as certainty of the evidence was very low [36]. Additionally, the authors showed low-certainty evidence that maintenance with propofol may reduce POCD [36]. Thus, there is insufficient evidence to inform the choice of general anesthetic agent with respect to the beneficial effect during surgery in the elderly patients.

It was well known that volatile anesthetics were able to impair insulin secretion and glucose utilization [37]. Our previous studies using patch clamp experiments and intravenous glucose tolerance tests in rabbits indicated that isoflurane-induced inhibition of insulin secretion was mediated by the isoflurane-induced opening of adenosine triphosphate-sensitive potassium (K_ATP_) channels in pancreatic β-cells, while propofol had no effects on the K_ATP_ channels in pancreatic β-cells, consequently no inhibition of insulin secretion [38, 39]. It is also reported that sevoflurane reduced glucose tolerance compared with propofol [40]. In the present study, we showed in the elderly patients that low-dose glucose load was able to be safe during sevoflurane based anesthesia. These results suggest that low-dose glucose load may be safe during propofol based anesthesia as well.

There were no significant differences in EE and RQ data in the current study of elderly patients or our previous study of adult subjects [12]. The low-dose glucose load may, therefore, not influence EE and RQ, although these parameters may change in patients undergoing major surgery.

This study has several limitations. First, data were obtained over a relatively short period, the final timepoint being the morning of postoperative day 1. Although we did not investigate the influence of glucose on long-term outcomes, there were no significant differences in protein catabolism. Second, in present study, the surgeries were minor surgery, because we want to exclude the influence of surgical stress to see the effect of aging on lipid metabolism in the present study. There may be significantly difference in prolonged and major surgery. Further studies to see the differences of low-dose glucose loading on the lipid metabolism and protein catabolism in the prolonged and major surgery are needed. However, we demonstrated that even in minor surgery with a little stress, low-dose glucose loading to the elderly patients improved lipid metabolism without developing hyperglycemia. Prolonged and major surgery in the absence of glucose loading was shown to induce protein catabolism in adults [13]. These results suggest that in major surgeries and/or prolonged surgeries which induce more remarkable stress, low-dose glucose loading to the elderly patients may control the increase of FFA and the ketone bodies and inhibit protein catabolism, when compared without glucose loading. Third, there were differences in the types of surgical procedures performed on patients in the two groups. These differences, however, were regarded as irrelevant because the concentrations of ACTH and cortisol during surgery were similar in the two groups. Finally, in the present study, the elderly patients who were not diabetes were subject of the study. The effects of low dose glucose infusion on both glucose and fat metabolism on elderly patients with diabetes or acute neurologic insults are not clear from the findings of the present study.

## Conclusions

The present study indicates that intraoperative low glucose infusion during remifentanil-induced anesthesia attenuated the catabolism of fat without causing harmful hyperglycemia in this population of elderly patients. These data suggest that low-dose glucose loading may be useful in elderly patients undergoing relatively minor surgical procedures.

## Data Availability

The date and materials are available from the corresponding author upon reasonable request.

## Abbreviations

3-MH: 3-methylhistidine
ACTH: adrenocorticotropic hormone
Cr: creatinine
EE: energy expenditure
FFA: free fatty acid
HOMA-IR: Homeostasis Model Assessment Insulin Resistance
RQ: respiratory quotient
$$ \dot{\mathrm{V}} $$O_2_: oxygen consumption
$\dot{\mathrm{V}}$CO_2_: carbon dioxide output
